# Segmentation of partial least squares structural equation modelling using kernel K-means clustering (PLS SEM KKC)

**DOI:** 10.1016/j.mex.2025.103570

**Published:** 2025-08-18

**Authors:** Cindy Cahyaning Astuti, Bambang Widjanarko Otok, Shofi Andari

**Affiliations:** a Department of Statistics, Faculty of Science and Data Analytics, Institut Teknologi Sepuluh Nopember, Surabaya 60111, Indonesia; b Faculty of Psychology and Education, Universitas Muhammadiyah Sidoarjo, Sidoarjo 61215, Indonesia

**Keywords:** Segmentation, PLS SEM, Kernel K-Means Clustering

## Abstract

This study proposes a new method in PLS SEM segmentation, namely PLS SEM Kernel K-Means Clustering (PLS SEM KKC). Segmentation is conducted to overcome one of the main limitations of PLS SEM modeling: unobserved heterogeneity. Previous studies on segmentation in PLS SEM have been performed using a linear clustering method. Segmentation is carried out based on the residual values of measurement and structural models from global PLS SEM, where the characteristics of the residuals are non-linear; thus, the segmentation process requires non-linear segmentation. This study's primary contribution is integrating kernel-based clustering into PLS SEM segmentation. The method effectively addresses unobserved heterogeneity by capturing non-linear residual patterns, leading to more accurate models. The empirical results show that the PLS SEM KKC method significantly improves model accuracy, with R² increasing from 51.1 % (global model) to 93.9 % (*k* = 2) and 97.5 % (*k* = 3) in segmented clusters. The increase in local R² confirms overcoming unobserved heterogeneity by grouping observations with similar patterns into homogeneous segments, improving model accuracy.

• This study recommends a new method in PLS SEM segmentation.

• PLS SEM KKC effectively captures non-linear residual patterns to address unobserved heterogeneity and improve model accuracy.

## Graphical abstract

**Figure:**
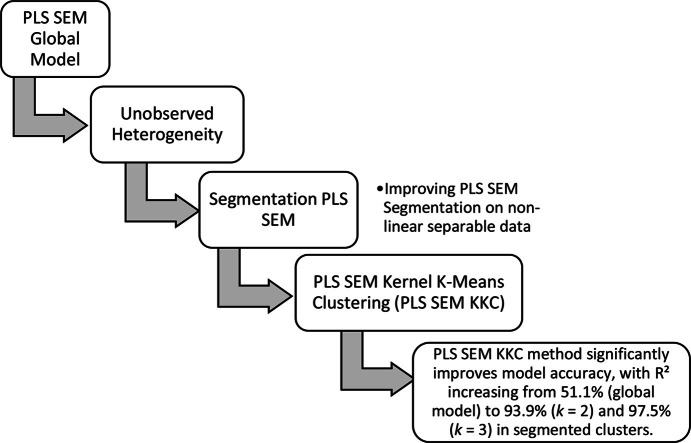

## Specifications table

**Table:** 

**Subject area**	Mathematics and Statistics
**More specific subject area**	Statistics; Multivariate Analysis; Segmentation PLS SEM
**Name of your method**	Partial Least Square Structural Equation Modelling (PLS SEM KKC)
**Name and reference of original method**	M. Fordellone and M. Vichi, “Finding Groups in Structural Equation Modeling Through The Partial Least Squares Algorithm,” *Computational Statistics Data Analysis*, vol. 147, Jul. 2020, doi:10.1016/j.csda.2020.106957.
**Resource availability**	Data on students' behavioral intention to use e-learning is available on request.

## Background

Partial Least Squares Structural Equation Modeling (PLS SEM) is a technique used to model the relationship between latent variables in a conceptual model [1]. The limitations of PLS SEM modeling include unobserved heterogeneity as one of the main factors. PLS SEM data contains unobserved heterogeneity when variations occur in data sources that remain unmeasurable due to latent variables. The approach of PLS SEM produces inaccurate parameter measurements when unobserved respondent variation goes unnoticed because the system fails to recognize differences between its users. The segmentation process serves as a solution for overcoming unobserved heterogeneity problems in PLS SEM through clustering analysis to segment the data [2].

Research on unobserved heterogeneity in PLS SEM modeling has been conducted using Finite Mixture PLS [3] and segmentation PLS SEM using hierarchy clustering analysis known as REBUS PLS [4]. The development of segmentation in PLS SEM was also carried out using K-Means clustering analysis with segmentation orientation as the latent variable score [5]. Previous research using PLS SEM segmentation applied the linear clustering method to implement its clustering procedures with explicit straight-line separations. The linear clustering process fails to produce optimal results when data contains properties that cannot be divided linearly through straight lines. The study introduces Kernel K-means Clustering as a new segmentation option for PLS SEM to manage data that cannot be separated linearly, hereinafter referred to as PLS SEM KKC. The kernel functions transform data into extended dimensions where non-linear patterns can be easily detected to separate non-linearly separable patterns. The implementation of PLS SEM KKC is expected to enhance accuracy for more effective result representation.

This approach applies the PLS SEM model segmentation through the Kernel K-Means clustering technique to manage unobserved heterogeneity through grouping sample units based on PLS SEM model residuals. The data consists of residuals that usually show the non-linear separation patterns. A model based on PLS SEM produces good results when its residuals appear near zero while other residuals remain distant from zero, thus demonstrating non-linear separability. The globular pattern indicates non-linear separability of this residual data, so the Kernel K-Means Clustering method is needed to perform separation. The homogeneous groups created through Kernel K-Means Clustering improve the accuracy and validity of the PLS SEM model, which will be built. The local PLS SEM model achieves better accuracy than the global model based on R^2^ measurement results from segmentation. The clustering density evaluation in segmentation relies on the Calinski-Harabasz (CH) Index for assessment [6]. The alternative modeling approach is used to analyze student behavioral intentions according to UTAUT, followed by performance testing on the developed PLS SEM KKC method through simulation analysis.

## Method details

### Partial least squares structural equation modeling (PLS SEM)

PLS SEM is a statistical analysis technique used to model the relationship between latent variables. PLS SEM employs a regression approach using the OLS (Ordinary Least Squares) method in the estimation process [7]. PLS SEM is designed to maximize the predictive power of the model by explaining as much variance as possible in the dependent variable used. Partial least squares (PLS) is a powerful analytical method and is often referred to as soft modeling because it eliminates the assumptions of the OLS technique, such as the distribution of residuals not having to be a multivariate normal distribution. PLS SEM is popularly used as an analytical method in various fields such as health [8], education [9], and socio-economic fields [10]. PLS SEM analysis consists of three stages, namely assessing the measurement results of the reflective model (outer model), assessing the measurement results of the formative model (outer model), and assessing the measurement results of the structural model (inner model) [11]. The reflective model is used when variables are considered as manifestations of a construct. These variables are seen as indicators that reflect the same properties of the construct being measured. In a reflective model, variables are considered to be related to the underlying construct, and changes in the construct affect changes in the variables. The reflective model considers the latent variable as the cause of the indicator, so it is called reflective because the indicator reflects the latent variable [12]. Meanwhile, the formative model considers that the indicator causes the latent variable. Formative models are used when variables are regarded as factors that shape or influence a construct. In the formative model, variables are considered as constituents or determinants of the construct being measured [13]. The estimation process of variance-based SEM or PLS models consists of weight estimation and path estimation. Weight estimate is an iterative procedure that considers the inner model, outer model, and weight relations to obtain factor scores by estimating latent variables. Since the latent variable values are not known with certainty, weight relations must be defined to complete the latent variable estimation process. Weight relations are weights to estimate latent variables as a linear combination of each indicator [14].

The measurement model or outer model, shows how each indicator relates to its latent variable which is measured based on the dimensions that form a factor in the indicator [15]. The reflective measurement model (outer model reflective) can be expressed as follows:

(1) $$\underset{A\times 1}{x}=\underset{A\times p}{\Lambda_{x}}\underset{p\times 1}{\xi }+\underset{A\times 1}{\delta_{x}}$$

(2) $$\underset{B\times 1}{y}=\underset{B\times q}{\Lambda_{y}}\underset{q\times 1}{\eta }+\underset{B\times 1}{{ɛ}_{y}}$$

x = indicator vector for exogenous latent variable constructs (ξ) of size A×1.

y = indicator vector for endogenous latent variable constructs (η) of size B×1.

$\Lambda_{x}$= loading matrix for the coefficient connecting the exogenous latent variable with the indicator of size A×p.

$\Lambda_{y}$= loading matrix for the coefficient connecting the endogenous latent variable with the indicator of size B×p.

$\delta_{x}$ = measurement error residuals for exogenous latent variables of size A×1.

${ɛ}_{y}$ = measurement error residuals for endogenous latent variables of size B×1.

*A* = total number of indicators on exogenous latent variables $\left(\sum a_{p}=A;p=1,2,...,p\right)$, where *p* is the number of indicators of exogenous variables.

*B* = total number of indicators on endogenous latent variables $\left(\sum b_{q}=B;q=1,2,...,q\right)$, where *q* is the number of indicators of exogenous variables.

The measurement model of exogenous latent variables with *p* exogenous latent variables and the total number of indicators as many as *A* based on equations (1) can be written in a matrix-shaped equation as follows:

$$\underset{A\times 1}{\left[\begin{array}{c} x_{11}\\ x_{12}\\ \vdots \\ x_{1a_{1}}\\ x_{21}\\ x_{22}\\ \vdots \\ x_{2a_{2}}\\ \vdots \\ x_{p1}\\ x_{p2}\\ \vdots \\ x_{pa_{p}} \end{array}\right]}=\underset{A\times p}{\left[\begin{array}{cccc} \lambda_{11} & 0 & \cdots & 0\\ \lambda_{12} & 0 & \cdots & 0\\ \vdots & \vdots & \ddots & \vdots \\ \lambda_{1a_{1}} & 0 & \cdots & 0\\ 0 & \lambda_{21} & \cdots & 0\\ 0 & \lambda_{22} & \cdots & 0\\ \vdots & \vdots & \ddots & \vdots \\ 0 & \lambda_{2a_{2}} & \ldots & 0\\ \vdots & \vdots & \ddots & \vdots \\ 0 & 0 & \cdots & \lambda_{p1}\\ 0 & 0 & \cdots & \lambda_{p2}\\ \cdots & \cdots & \ddots & \vdots \\ 0 & 0 & \cdots & \lambda_{pa_{p}} \end{array}\right]}\underset{p\times 1}{\left[\begin{array}{c} \xi_{1}\\ \xi_{2}\\ \vdots \\ \xi_{p} \end{array}\right]}+\underset{A\times 1}{\left[\begin{array}{c} \delta_{11}\\ \delta_{12}\\ \vdots \\ \delta_{1a_{1}}\\ \delta_{21}\\ \delta_{22}\\ \vdots \\ \delta_{2a_{2}}\\ \vdots \\ \delta_{p1}\\ \delta_{p2}\\ \vdots \\ \delta_{pa_{p}} \end{array}\right]}$$

The measurement model equation based on endogenous latent variables with endogenous latent variables is one and the total number of indicators is B based on Eq. (2) can be written in matrix form as follows:

$$\underset{B\times 1}{\left[\begin{array}{c} y_{1}\\ \vdots \\ y_{q} \end{array}\right]}=\underset{B\times 1}{\left[\begin{array}{c} \lambda_{1}\\ \vdots \\ \lambda_{q} \end{array}\right]}\underset{1x1}{\left[\eta \right]}+\underset{B\times 1}{\left[\begin{array}{c} \varepsilon_{1}\\ \vdots \\ \varepsilon_{q} \end{array}\right]}$$

### Kernel K-means clustering

Kernel K-means Clustering is an extension of the K-means algorithm that utilizes a Kernel function to transform the high-dimensional data mapping into a new space, thus allowing for a linear separation of the data. This method aims to select starting points from denser regions because these regions reflect the nature of the entire data set [16]. The clustering process using Kernel K-means is a development of the K-means algorithm that utilizes the Kernel method to map high-dimensional data into a new space, allowing for linear separation. The Kernel K-Means Clustering method calculates the initial seed value in the K-means clustering algorithm using the kernel algorithm. The goal is to increase the accuracy of the clustering results. In the Kernel K-means method, it is expected that the data can be separated better, so that if there is an overlap between data, it can be arranged to be linear in a new dimension. The conversion of the K-Means method into the Kernel K-Means method is realized by changing the concept of distance into the form of a Kernel function [17].

The *K* function can be defined as a Kernel function that on all input vectors, can be expressed in the equation:

$$K\left(u_{c},v_{i}\right)=\phi {\left(u_{c}\right)}^{T}\phi \left(v_{i}\right)$$

The mapping function from input space to feature space is expressed by ϕ(.) or mathematically can be written as ϕ:u→ϕ(v)∈F. The use of kernel functions allows the use of a model feature space without the need to define from the input space to the feature space [18]. Some Kernel functions can be expressed in the following equation:

1. Linear Kernel Function
  $$K\left(u_{c},v_{i}\right)=\left(u_{c},v_{i}\right)$$
2. Polynomial Kernel Function
  $$K_{pol}\left(u_{c},v_{i}\right)={\left(u_{c}.v_{i}+\tau \right)}^{\theta }$$
3. Gaussian Kernel Function / *Radial Basic Function* (RBF)
  $$K_{RBF}\left(u_{c},v_{i}\right)=\exp\left(-\frac{{∥u_{c}-v_{i}∥}^{2}}{2\sigma^{2}}\right)=\exp\left(-\kappa \left({∥u_{c}-v_{i}∥}^{2}\right)\right)$$
4. Sigmoid Kernel Function
  $$K_{sig}\left(u_{c},v_{i}\right)=\tan\left(\theta u_{c}.v_{i}+\tau \right)$$

Based on several types of kernel functions, namely linear, polynomial, RBF and sigmoid kernels, the RBF kernel function has several advantages compared to other kernel functions. Among others, the RBF kernel can map input data into a high-dimensional feature space where data that was originally non-linearly separable in the original space becomes linearly separable in high-dimensional space. It allows the model to overcome data complexity and become a better separator in the clustering process [19]. The RBF kernel is effective in handling data with non-linear relationships because the shape of the Gaussian function creates the necessary complexity. The Gaussian function underlying the RBF kernel provides flexibility in forming decision boundaries that can adjust to the complexity and non-linearity of the data. It makes the RBF kernel effective in clustering non-linearly separable data. The RBF kernel generally has fewer parameters compared to other kernel functions [20]. Parameterκdetermining the width of the Gaussian function is generally considered simpler in terms of the number of parameters that need to be adjusted compared to other kernel functions, such as polynomial kernel functions that require determining the degree of the polynomial [21]. It makes the RBF kernel easier to use on various types of data. Under certain conditions the RBF kernel can be a universal approximator, meaning that with proper parameter adjustment and sufficient data, the RBF kernel can approximate any target function with the desired accuracy. It means that the RBF kernel can capture very complex relationships in data [22]. Given its advantages, the RBF kernel function is the one employed in this research.

### Unified theory of acceptance and use of technology (UTAUT)

The development of information technology systems is growing rapidly in the field of education. One form of utilization of information technology, especially internet technology in the field of education is the use of e-learning. E-learning is electronic learning that refers to the use of information and communication technology in a learning process. E-learning involves the use of computers, the internet, mobile devices, and online learning platforms to deliver learning materials and facilitate interaction between learners and teachers. In order for an e-learning system that has been made to be well accepted by its users, it is necessary to have a model that can describe the acceptance of an information technology system that has been made [23]. Research was conducted to examine the theory related to e-learning acceptance by students as users using the Unified Theory of Acceptance and Use of Technology (UTAUT) model. The UTAUT model was developed to understand the acceptance and use of information technology by users [24]. This study adopts the UTAUT 1 Model, focusing on four variable constructs, namely Performance Expectancy (PE), Effort Expectancy (EE), Social Influence (SI), and Facilitating Conditions (FC) which affect Behavioral Intention (BI) [25]. The measure of performance expectancy establishes how much people believe their system usage delivers advantages to their activities. Some users need to understand how easy it is to employ a system or technology through the effort expectancy variable [26]. Social influence measures how much weight an individual attaches to the perceived significance that influential others believe regarding implementing new technology. The evaluation of available technical and organizational support systems enables the determination of facilitating conditions for system or technology adoption [27]. The UTAUT model has been used in various contexts to understand user acceptance of technology, including acceptance of e-learning [28], information systems, mobile devices, e-commerce, and others. Several studies in the field of education have been conducted with the UTAUT model. In the field of education, PLS SEM analysis using the UTAUT model can provide an in-depth understanding of the factors that influence the acceptance and use of technology in the learning process [29].

**Fig. 1:**
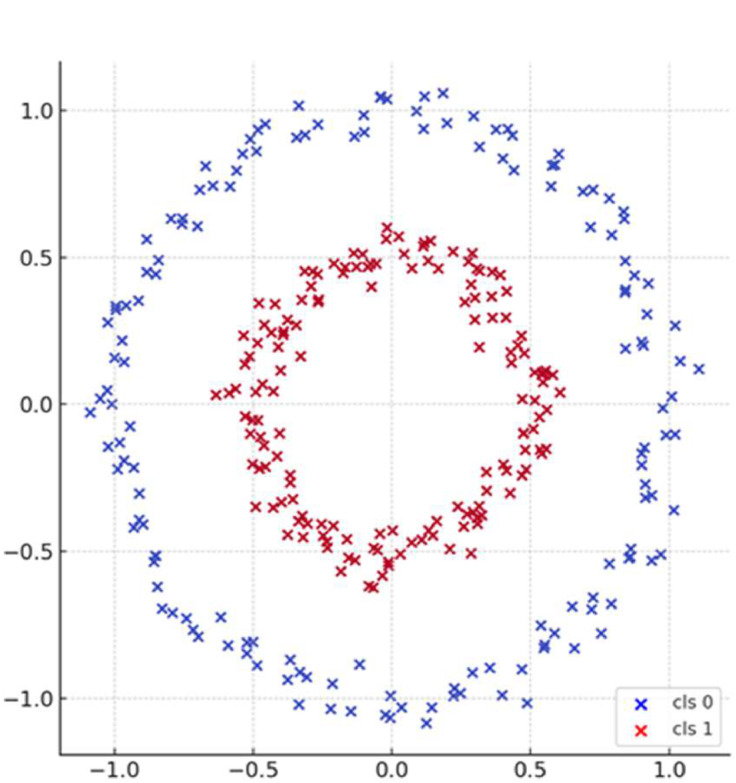
Residual data subjected to a non-linear clustering using KKC.

**Fig. 2:**
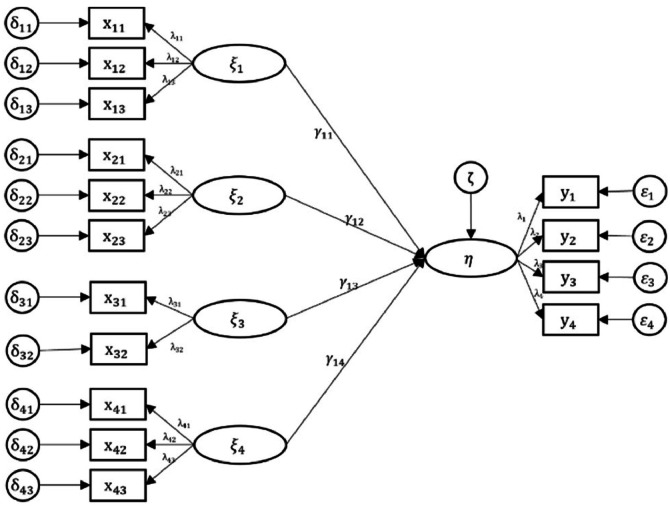
Research conceptual model.

Segmentation in PLS SEM Kernel K-means Clustering (PLS SEM KKC) is performed using residual data from Global PLS SEM modeling. In the clustering process using Kernel K-means, residual values that are small or close to zero tend to form one segment, while residuals that are large or far from zero form another segment. The residual value can show how well the PLS-SEM model is able to represent the data. The residual value in the context of PLS-SEM (is the difference between the actual (observed) value and the value predicted by the model. If the residual has a small value or is close to zero, it indicates that the model prediction is very close to the observed value, which means that the model has a high level of representation and fit to the data. Conversely, if the residuals have a large value or are far from zero, it indicates a significant deviation between the model prediction and the actual data. The large deviation reflects that at certain data points, the model does not correctly capture the pattern or structure that exists in the data, resulting in decreased prediction accuracy. Kernel K-means Clustering takes into account non-linear relationships through a feature space transformation suitable for residual data patterns [30]. PLS SEM KKC segmentation can detect the cluster structure in non-linear data by using kernel function clusters that cannot be linearly separated become separated in the new feature space. Therefore residuals with similar characteristic values can be grouped optimally. The following is an illustration of residual data subjected to a non-linear clustering process using Kernel K-means Clustering in Fig. 1:

The purpose of this study is to obtain the PLS SEM KKC algorithm to overcome unobserved heterogeneity. To begin the process, use the UTAUT model for determining structural equation models and measurement models between latent variables. The conceptual model of this study consists of one endogenous latent variable and 4 exogenous latent variables where each exogenous latent variable has 3, 3, 2 and 3 indicators, while the endogenous latent variable has 4 indicators, which can be depicted in Fig. 2:

Structural model equation formed based on Fig. 2:

$$\eta =\gamma_{11}\xi_{1}+\gamma_{12}\xi_{2}+\gamma_{13}\xi_{3}+\gamma_{14}\xi_{4}+\zeta$$

The data used in the segmentation process is data based on the residual of the measurement model and structural model residuals from the PLS SEM analysis based on Eq. (3).

(3) $$R*=\left[\delta ,ɛ,\zeta \right]=\left[\begin{array}{cccccccccccccccc} \delta_{1,11} & \delta_{1,12} & \delta_{1,13} & \delta_{1,21} & \delta_{1,22} & \delta_{1,23} & \delta_{1,31} & \delta_{1,32} & \delta_{1,41} & \delta_{1,42} & \delta_{1,43} & \varepsilon_{1,1} & \varepsilon_{1,2} & \varepsilon_{1,3} & \varepsilon_{1,4} & \zeta_{1,1}\\ \begin{array}{c} \delta_{3,11}\\ \delta_{3.11} \end{array} & \begin{array}{c} \delta_{2,12}\\ \delta_{3.12} \end{array} & \begin{array}{c} \delta_{2,13}\\ \delta_{3,13} \end{array} & \begin{array}{c} \delta_{2,21}\\ \delta_{3,21} \end{array} & \begin{array}{c} \delta_{2,22}\\ \delta_{3,22} \end{array} & \begin{array}{c} \delta_{2,23}\\ \delta_{3,23} \end{array} & \begin{array}{c} \delta_{2,31}\\ \delta_{3,31} \end{array} & \begin{array}{c} \delta_{2,32}\\ \delta_{3,32} \end{array} & \begin{array}{c} \delta_{2,41}\\ \delta_{3,41} \end{array} & \begin{array}{c} \delta_{2,42}\\ \delta_{3,42} \end{array} & \begin{array}{c} \delta_{2,43}\\ \delta_{3,43} \end{array} & \begin{array}{c} \varepsilon_{2,1}\\ \varepsilon_{3,1} \end{array} & \begin{array}{c} \varepsilon_{2,2}\\ \varepsilon_{3,2} \end{array} & \begin{array}{c} \varepsilon_{2,3}\\ \varepsilon_{3,3} \end{array} & \begin{array}{c} \varepsilon_{2,4}\\ \varepsilon_{2,4} \end{array} & \begin{array}{c} \zeta_{3,1}\\ \zeta_{3,1} \end{array}\\ \vdots & \vdots & \vdots & \vdots & \vdots & \vdots & \vdots & \vdots & \vdots & \vdots & \vdots & \vdots & \vdots & \vdots & \vdots & \vdots \\ \delta_{n,11} & \delta_{n,12} & \delta_{n,3} & \delta_{n,21} & \delta_{n,22} & \delta_{n,23} & \delta_{n,31} & \delta_{n,32} & \delta_{n,41} & \delta_{n,42} & \delta_{n,43} & \varepsilon_{n,1} & \varepsilon_{n,2} & \varepsilon_{n,3} & \varepsilon_{n,4} & \zeta_{n,1} \end{array}\right]$$

Residual value of the measurement model and the residual structural model resulting from the PLS SEM analysis in Eq. (3) is redefined as **R** and written in Eq. (4):

(4) $$R=\left[\begin{array}{cccccccc} e_{1,1} & e_{1,2} & e_{1,3} & e_{1,4} & e_{1,5} & e_{1,6} & \ldots & e_{1,S}\\ e_{2,1} & e_{2,2} & e_{2,3} & e_{2,4} & e_{2,5} & e_{2,6} & \ldots & e_{2,S}\\ e_{3,1} & e_{3,2} & e_{3,3} & e_{3,4} & e_{3,5} & e_{3,6} & \ldots & e_{3,S}\\ e_{4,1} & e_{4,2} & e_{4,3} & e_{4,4} & e_{4,5} & e_{4,6} & \ldots & e_{4,S}\\ e_{5,1} & e_{5,2} & e_{5,3} & e_{5,4} & e_{5,5} & e_{5,6} & \ldots & e_{5,S}\\ e_{6,1} & e_{6,2} & e_{6,3} & e_{6,4} & e_{6,5} & e_{6,6} & \ldots & e_{6,S}\\ \vdots & \vdots & \vdots & \vdots & \vdots & \vdots & \ddots & \vdots \\ e_{n,1} & e_{n,2} & e_{n,3} & e_{n,4} & e_{n,5} & e_{n,6} & \ldots & e_{n,S} \end{array}\right]$$

The stages PLS SEM KKC based on the residual measurement structural model in PLS SEM modelling. The kernel function used is the Gaussian kernel or RBF based on Eq. (5):

(5) $$K\left(e_{i},e_{i}\right)=\exp\left(-\frac{{∥e_{i}-e_{i}∥}^{2}}{2\sigma^{2}}\right)=\exp\left(-\kappa \left({∥e_{i}-e_{i}∥}^{2}\right)\right)$$

In this research, kernel parameter for the RBF was determined by setting the kernel width σ=1which corresponds to kernel parameterκ=0.5 in the RBF formulation. The value was chosen as a commonly used baseline in kernel based clustering studies, providing a balanced trade-off between model flexibility and generalization. It allows the algorithm to capture local non-linear structures without leading to overfitting. The kernel matrix is a symmetric matrix with size n×n according to the number of residual units that are clustering. Then, the Kernel K-Means matrix based on Eq. (5) can be calculated. Kernel matrix with size n×ncan be written in Eq. (6):

(6) $$K\left(e_{i},e_{i}\right)=\left[\begin{array}{ccccccc} K(e_{1},e_{1}) & K(e_{1},e_{2}) & K(e_{1},e_{3}) & K(e_{1},e_{4}) & K(e_{1},e_{5}) & \ldots & K(e_{1},e_{n})\\ K(e_{2},e_{1}) & K(e_{2},e_{2}) & K(e_{2},e_{3}) & K(e_{2},e_{4}) & K(e_{2},e_{5}) & \ldots & K(e_{2},e_{n})\\ K(e_{3},e_{1}) & K(e_{3},e_{2}) & K(e_{3},e_{3}) & K(e_{3},e_{4}) & K(e_{3},e_{5}) & \ldots & K(e_{3},e_{n})\\ K(e_{4},e_{1}) & K(e_{4},e_{2}) & K(e_{4},e_{3}) & K(e_{4},e_{4}) & K(e_{4},e_{5}) & \ldots & K(e_{4},e_{n})\\ K(e_{5},e_{1}) & K(e_{5},e_{2}) & K(e_{5},e_{3}) & K(e_{5},e_{4}) & K(e_{5},e_{5}) & \ldots & K(e_{5},e_{n})\\ \vdots & \vdots & \vdots & \vdots & \vdots & \ddots & \vdots \\ K(e_{n},e_{1}) & K(e_{n},e_{2}) & K(e_{n},e_{3}) & K(e_{n},e_{4}) & K(e_{n},e_{5}) & \ldots & K(e_{n},e_{n}) \end{array}\right]$$

In the segmentation stage using Kernel K-means Clustering, two initialization scenarios were performed .The number of clusters was pre-specified as *k* = 2 and *k* = 3 to reflect typical segmentation scenarios and to facilitate comparative analysis After the initial initialization is complete, the next step is to calculate the kernel distance for each data. Kernel distance is calculated by referring to the clusters that have been determined based on Eq. (7):

(7) $$d\left(i,k\right)=K\left(e_{i},e_{i}\right)-\frac{2}{n_{k}}\sum_{l=1}^{n_{k}}K(e_{i},e_{l})+\frac{1}{{\left(n_{k}\right)}^{2}}\sum_{m=1}^{n_{k}}\sum_{l=1}^{n_{k}}K(e_{m},e_{l})$$

Cluster membership is formed from minimum kernel distance d(i,k) or it can be stated that $e_{i}$is included in the cluster that has the smallest d(i,k)value, according to following equation:

clustermembership=argmind(i,k)

After each data $e_{i}$is a member of the new cluster, the set of clustering initialization $e_{i}$ is updated. Then, repeat the kernel distance calculation based on the cluster membership formed. Iteration stops if there is no more data movement from one cluster to another. The next step is estimating the parameters of PLS SEM model in each segment formed in the local PLS SEM model. In each group of results from the segmentation process, PLS SEM KKC modeling is carried out to produce a local PLS SEM KKC model in each segment. The Calinski Harabasz (CH) index was used to gauge the quality of the segmentation results obtained with PLS SEM KKC. CH index is implemented to measure how well an object is in the group or cluster that has been done in clustering analysis based on Eq. (8):

(8) $$CH=\frac{Tr(B_{k})}{Tr(W_{k})}\times \frac{n-k}{k-1}$$

k = number of clusters.

$Tr(B_{k})$= trace from between cluster dispersion matrix.

$Tr(W_{k})$= trace from within cluster dispersion matrix.

Model evaluation using R^2^ on the global PLS SEM model and local PLS SEM model based on equation [9]:

(9) $$R_{j}^{2}=1-\frac{\sum_{i=1}^{n}\left(Y_{ji}-{\overset{⌢}{Y}}_{ji}\right)}{\sum_{i=1}^{n}\left(Y_{ji}-{\bar{Y}}_{ji}\right)}$$

Basically, Kernel K-means Clustering is the development of K-means Clustering by adding the concept of Kernel. The kernel technique makes it possible to map data on a high-dimensional feature space without performing explicit calculations. Each residual value $e_{i}$ is mapped to a point in the feature space $\phi (e_{i})$. The value of $\phi (e_{i})$does not need to be calculated explicitly but simply uses the kernel concept of $\left(\phi \left(e_{i}\right),\phi \left(e_{i}\right)\right)=K\left(e_{i},e_{i}\right)$. The use of kernel techniques helps to find cluster divisions that are more in line with the actual data structure. The determination of the kernel distance in the Kernel K-means Clustering algorithm is based on Eq. (7). It is described in Theorem 1 that the kernel distance calculation is obtained from the description of the kernel objective function defined in the feature space.

Theorem 1 Define the residuals of the results of PLS SEM modeling, namely $R=\{\begin{array}{ccccc} e_{i,1} & e_{i,2} & e_{i,3} & \ldots & e_{i,S} \end{array}\}$ the set of residuals resulting from SEM PLS modeling. The kernel matrix **K** with the following elements:

$$K\left(e_{i},e_{i}\right)=\exp\left(-\kappa {∥e_{i}-e_{i}∥}^{2}\right)$$

Grouping the residual data into *k* clusters k={1,2,...,K} with a kernel objective function:

$$J=\sum_{i=1}^{n}{∥\phi \left(e_{i}\right)-\mu_{k}∥}^{2}$$

Then, the minimum result of the kernel objective function can be presented based on Eq. (8):

$$J=K\left(e_{i},e_{i}\right)-\frac{2}{n_{k}}\sum_{l=1}^{n_{k}}K(e_{i},e_{l})+\frac{1}{{\left(n_{k}\right)}^{2}}\sum_{m=1}^{n_{k}}\sum_{l=1}^{n_{k}}K(e_{m},e_{l})$$

The study proposes a novel method PLS SEM KKC to handle unobserved heterogeneity by segmenting residuals from PLS SEM using kernel-based clustering. The following procedures are the summary of the PLS-SEM KKC segmentation process:

1. Model Specification
  Define structural and measurement models based on a conceptual framework (use UTAUT model).
2. Global PLS SEM Estimation
  Estimate path coefficients and obtain residuals from both measurement and structural models.
3. Residual Extraction
  Extract residual vectors based on Eq. (4) for each observation to form input for clustering.
4. Kernel K-Means Segmentation
  Apply Gaussian/RBF and calculate Kernel K-Means matrix based on Eq. (6) with kernel parameterκ=0.5.
  Compute kernel distance matrix based on Eq. (7) and perform clustering with *k* = 2 and *k* = 3.
  Evaluate Clustering with CH Index based on Eq. (8).
5. Local PLS SEM Modeling
  Re-estimate PLS SEM models separately for each cluster to assess R² improvement.
6. Evaluation
  Compare global and segmented model performance using R² based on Eq. (9).

**Fig. 3:**
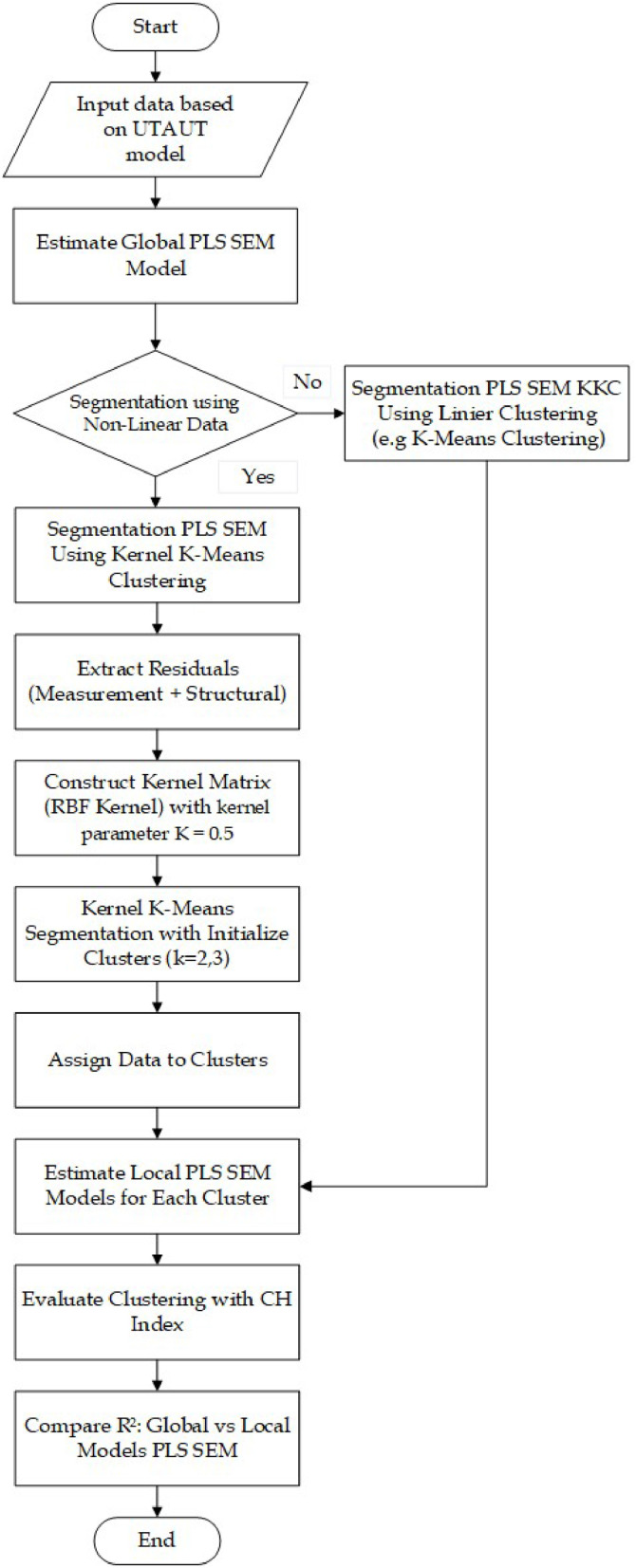
Flowchart of data analysis.

The flowchart of data analysis presented in Fig. 3:

The data analysis using PLS SEM KKC are presented in Fig. 3. PLS SEM KKC is the development of a new method in PLS SEM segmentation that uses non-linear clustering methods. PLS SEM KKC applied to students' behavioral intention to use e-learning at Universitas Muhammadiyah Sidoarjo. The data analysis procedures in this study have been published and can be accessed through the following link:

https://rpubs.com/cindycahyaningastuti/Segmentation_PLSSEMKKC

The simulation study is conducted to evaluate the performance of the developed method, namely PLS SEM KKC, and aims to determine how well the developed method is to segment or group based on the residual value obtained from PLS SEM modeling. The simulation design that are carried out considers several factors, including the number of data, the number of segments and elements other than the main diagonal in the covariance matrix of data with normal distribution. The simulation study design factors is presented in Table 1:

**Table 1:** Simulation study design factors.

Factor	Level
The amount of data in the simulation study	1. 50 (small)
	2. 200 (Medium)
	3. 1000 (Large)
Elements other than the main diagonal in the variance covariance matrix of data with normal distribution	1. 0.3 2. 0.5 3. 0.8
Number of segments	1. Number of Segments 2 (*k* = 2)
	2. Number of Segments 3(*k* = 3)

The simulation design in Table 4 was conducted to evaluate the performance of the PLS SEM KKC model under various conditions of sample size and covariance between different variables. Data were randomly generated with multivariate normal distribution based on three scenarios of covariance values of 0.3, 0.5, and 0.8 to fill the covariance variance matrix. These values were deliberately chosen to represent different levels of correlation structures among the observed indicators that form the exogenous latent variables in the PLS SEM model. Specifically 0.3 represents a low to moderate level of association among indicators, 0.5 represents a moderate correlation, and 0.8 represents a strong correlation between indicators. This variation is important because, in structural equation modeling, the degree of covariance among indicators directly affects the measurement quality of latent variables and the subsequent performance of the model. By incorporating these levels, intended to simulate realistic data conditions that range from weakly to strongly related indicators, and to examine how well the PLS SEM KKC method performs under such differing latent structures. Simulations were conducted for three sample sizes of n = 50 (small), 200 (medium), and 1000 (large). Furthermore, the data were analyzed using PLS SEM Global model, namely PLS SEM modeling without segmentation and PLS SEM KKC with 2 cluster segmentation (*k* = 2) and 3 cluster segmentation (*k* = 3). The algorithm for PLS SEM KKC Simulation is presented in Table 2:

**Table 2:** Algorithm for PLS SEM KKC simulation.

**Define**Number of exogenous latent variables$\xi_{p}$and number of endogenous latent variables η*p*: number of indicators of exogenous variables.*q*: number of indicators of endogenous variables.Assume • The true PLS SEM model structure follows the UTAUT framework with 4 exogenous latent variables (PE, EE, SI, FC) and 1 endogenous latent variable (BI). • Covariance among indicators is set at 0.3, 0.5, and 0.8 to reflect low, moderate, and high correlation scenarios. • Simulations were conducted for three sample sizes of *n* = 50 (small), 200 (medium), and 1000 (large) Data Generating Process
Generate exogenous indicators$\xi_{p}=\left[\begin{array}{cccc} \xi_{1,} & \xi_{2,} & \xi_{3,} & \xi_{4} \end{array}\right]∼N\left(0,\Sigma_{\xi }\right)$Compute latent variables • Calculate exogenous latent variables $\xi_{p}$​ using reflective measurement model. • Calculate endogenous latent variable ηusing structural model equation. • Extract residuals. Apply Kernel K-Means Clustering • Construct RBF kernel matrix with fixedκ=0.5. • Segment *k* = 2 and *k* = 3 cluster. • Re-estimate local PLS SEM. • For each cluster, re-calculate path coefficients. Evaluate • Evaluate clustering quality using Calinski-Harabasz (CH) Index. • Compute R² values for global and local models.

## Method validation

### Application of PLS SEM KKC on students' behavioral intention to use E-learning

PLS SEM KKC model is applied to the behavioral intention of students using e-learning at Universitas Muhammadiyah Sidoarjo, Indonesia. Data on students' behavioral intention to use e-learning is obtained based on the primary data from 366 students who are active users of e-learning. The conceptual model in this study uses the UTAUT model with four main constructs, namely performance expectancy (PE), effort expectancy (EE), social influence (SI), and facilitating conditions (FC). Each variable in the UTAUT conceptual model is measured through several indicators that reflect the variable being measured. The UTAUT model can be applied to the use of e-learning at Universitas Muhammadiyah Sidoarjo as an effort to improve the quality of education through a learning process that has a high level of flexibility.

Segmentation in PLS SEM using PLS SEM KKC is carried out with the number of 2 clusters (*k* = 2) and 3 clusters (*k* = 3) which produce local PLS SEM models in each segment formed. In addition, PLS SEM modeling is also carried out using the global model without the segmentation process. Furthermore, model evaluation is carried out using the R^2^ value on the global model and local model formed. Based on PLS SEM modeling, both the local PLS SEM KKC model and the global PLS SEM model, the structural model coefficient value is obtained which can be used to determine the effect of exogenous latent variables on endogenous latent variables presented in Table 3:

**Table 3:** Path coefficients global PLS SEM and PLS SEM KKC.

Variable	Global PLS SEM				PLS SEM KKC (*k* = 2)		PLS SEM KKC (*k* = 3)		
				Segment 1		Segment 2	Segment 1	Segment 2	Segment 3
Performance Expectancy (PE)		0.180	0.011			0.178	0.173	0.080	0.010
Effort Expectancy (EE)	-0.057		0.036			-0.075	-0.091	0.075	0.188
Social Influence (SI)	0.210		0.204			0.217	0.229	0.352	-0.048
Facilitating Conditions (FC)	0.507		0.762			0.457	0.469	0.555	0.856

Based on the structural model coefficients in Table 3, the structural model equations for PLS SEM in the global model and PLS SEM KKC in the local model and are as follows:

**Global PLS SEM Structural Model**

BI=0.180PE−0.057EE+0.210SI+0.507FC

**PLS SEM KKC (*k* = 2)**

PLS SEM KKC structural model with 2 clusters in segment 1

BI=0.011PE+0.036EE+0.204SI+0.726FC

PLS SEM KKC structural model with 2 clusters in segment 2

BI=0.178PE−0.075EE+0.217SI+0.457FC

**PLS SEM KKC (*k* = 3)**

PLS SEM KKC structural model with 3 clusters in segment 1

BI=0.173PE−0.0091EE+0.229SI+0.469FC

PLS SEM KKC structural model with 3 clusters in segment 2

BI=0.080PE+0.075EE+0.352SI+0.555FC

PLS SEM KKC structural model with 3 clusters in segment 3

BI=0.010PE+0.188EE−0.048SI+0.856FC

The quality of the segmentation results using PLS SEM KKC was measured using the Calinski Harabasz (CH) index. CH index is used to measure how well an object is in the group or cluster that has been done in the clustering analysis. The greater the CH index, the better the quality of the resulting clustering. The CH index results for PLS SEM KKC segmentation at *k* = 2 and *k* = 3 are presented in Table 4:

**Table 4:** Quality index of clustering result.

Number of Clusters	Quality Index of Clustering Result
	Calinski Harabasz (CH)
Number of Clusters 2 (*k* = 2)	13.84586
Number of Clusters 3 (*k* = 3)	8.042779

Based on the analysis results in Table 4, the CH value for the number of clusters 2 (*k* = 2) is 13.846 and the CH value for the number of clusters 3 (*k* = 3) is 8.043. The greater the CH Index value, the better the quality of the resulting clustering. The largest CH value is obtained in PLS SEM KKC segmentation with the number of clusters 2, so it can be said that the best quality of cluster results is PLS SEM KKC segmentation with the number of clusters 2 (*k* = 2).

Based on the analysis results in Table 5, the R² value of the PLS SEM KKC model shows a significant increase in several segments, indicating that the segmentation of PLS SEM based on Kernel K-Means Clustering (PLS SEM KKC) has succeeded in grouping data into a better structure than the global PLS SEM model. In the global PLS SEM model, the R² value is 51.1 %. Furthermore, when the residual data from the local PLS SEM model uses PLS SEM KKC with two segments (*k* = 2) the R² value in Segment 1 increased significantly to 93.9 %, while in Segment 2 it decreased to 40.2 %. Furthermore, when the number of Segments was increased to three Segments (*k* = 3), the R² value increased significantly in Segment 2 at 83.7 % and Segment 3 at 97.5 %, while in Segment 1 the R² value decreased to 41.9 %. The difference in R² values between Segments indicates the ability of PLS SEM KKC modeling to explain the variance in each data group. Kernel K-Means Clustering-based PLS SEM segmentation is performed using the residual value of the PLS SEM global modeling results, where data with residuals close to zero are considered the most representative groups.

**Table 5:** Significance level (p-value) of Global PLS SEM and PLS SEM KKC.

Variable	Global PLS SEM	PLS SEM KKC (*k* = 2)			PLS SEM KKC (*k* = 3)		
		Segment 1	Segment 2	Segment 1		Segment 2	Segment 3
Performance Expectancy (PE)	0.019*	0.871	0.014*	0.023*		0.542	0.786
Effort Expectancy (EE)	0.230	0.062	0.211	0.163		0.328	0.126
Social Influence (SI)	0.008*	0.017*	0.008*	0.006*		0.006*	0.360
Facilitating Conditions (FC)	0.000*	0.000*	0.000*	0.000*		0.013*	0.000*
R^2^	51.1 %	93.9 %	40.2 %	41.9 %		83.7 %	97.5 %

Significant at α = 5 %

The difference in R² values between segments illustrates the variation in the quality of the model in explaining the relationship between variables in each group. Kernel K-Means Clustering (KKC) segmentation using residuals allows the separation of data into groups with similar residual patterns. A good residual is close to zero, indicating that the model is able to predict with high accuracy the clustered data. Segments with significantly increased R² values, such as Segment 1 at *k* = 2 with an R² value of 93.9 % and Segment 3 at *k* = 3 with an R^2^ value of 97.5 %, are the result of grouping data with low or near-zero residuals. Conversely, groups with decreasing R² values indicate that the data in these segments have larger residuals, indicating that the model is less able to explain the relationship between variables in these groups. Therefore, an increase in the R² value can be interpreted as evidence of the segmentation's success in identifying groups with good predictive quality and being able to cluster data in groups with a stronger relationship structure.

### Simulation study

In the PLS SEM KKC segmentation approach, the segmentation process is carried out first, then each cluster is analyzed separately using PLS SEM which produces a local PLS SEM model in each segment. Simulation studies were conducted to see how the combination of sample size and covariance level affected R^2^value as an indicator of goodness of the model in explaining data variance. The combinations of nine simulations based on three different covariance values and three different sample sizes are presented in Table 6:

**Table 6:** Simulation design.

Factor	Covariance Values	Sample Size
Simulation 1	0.3	50
Simulation 2	0.3	200
Simulation 3	0.3	1000
Simulation 4	0.5	50
Simulation 5	0.5	200
Simulation 6	0.5	1000
Simulation 7	0.8	50
Simulation 8	0.8	200
Simulation 9	0.8	1000

The quality of the segmentation results in the simulation study was also measured using the Calinski Harabasz (CH) index. CH index is used to measure how well an object is in the group or cluster that has been done in the clustering analysis. The greater the CH value, the better the quality of the resulting clustering. The results of the CH index for the nine simulation studies are presented in Table 7:

**Table 7:** Quality index of clustering result.

Simulation Design	Quality Index of Clustering Result Calinski Harabasz (CH)	
	*k* = 2	*k* = 3
Simulation 1	0.966	0.716
Simulation 2	0.572	1.018
Simulation 3	10.560	15.034
Simulation 4	0.770	0.802
Simulation 5	1.124	2.179
Simulation 6	21.573	20.153
Simulation 7	2.292	2.039
Simulation 8	13.722	12.144
Simulation 9	75.398	64.215

Based on the analysis results in Table 7, there are variations in CH values in the nine simulation designs carried out with the number of clusters (*k*) of 2 and 3. Of all the simulations, there are three simulations that show the highest CH index based on 3 levels of covariance:

1. Simulation 3 (covariance level=0.3; *n* = 1000) the highest CH index for *k* = 3 is 15.034
2. Simulation 6 (covariance level=0.5; *n* = 1000) the highest CH index for *k* = 2 is 21.573
3. Simulation 9 (covariance level=0.8; *n* = 1000) the highest CH index for *k* = 2 is 75.398

These values show that the best models are found in Simulation 3, Simulation 6, and Simulation 9, each of which has a sample size of *n* = 1000. It indicates that larger sample sizes of *n* = 1000 tend to produce more stable and high-quality clustering results, as reflected by significantly higher CH values compared to other simulations.

Based on nine simulation study designs, the value of the structural model coefficient and significance value on the global PLS SEM model and the segmentation results of the local PLS SEM KKC with the number of Clusters 2 (*k* = 2) and the number of Clusters 3 (*k* = 3) are presented in Table A1, Table A2. The structural model equation for PLS SEM KKC local model on the best model based on CH index:

PLS SEM KKC structural model on Simulation 3 in Segment 1

BI=0.303PE+0.245EE+0.191SI+0.206FC

PLS SEM KKC structural model on Simulation 3 in Segment 2

BI=0.253PE+0.252EE+0.168SI+0.212FC

PLS SEM KKC structural model on Simulation 3 in Segment 3

BI=0.298PE+0.294EE+0.205SI+0.179FC

PLS SEM KKC structural model on Simulation 6 in Segment 1

BI=0.178PE+0.332EE+0.178SI+0.248FC

PLS SEM KKC structural model on Simulation 6 in Segment 2

BI=0.236PE+0.245EE+0.183SI+0.339FC

PLS SEM KKC structural model on Simulation 9 in Segment 1

BI=0.293PE+0.259EE+0.119SI+0.316FC

PLS SEM KKC structural model on Simulation 9 in Segment 2

BI=0.291PE+0.274EE+0.166SI+0.255FC

PLS SEM KKC structural model on Simulation 9 in Segment 3

BI=0.276PE+0.295EE+0.163SI+0.254FC

**Fig. 4:**
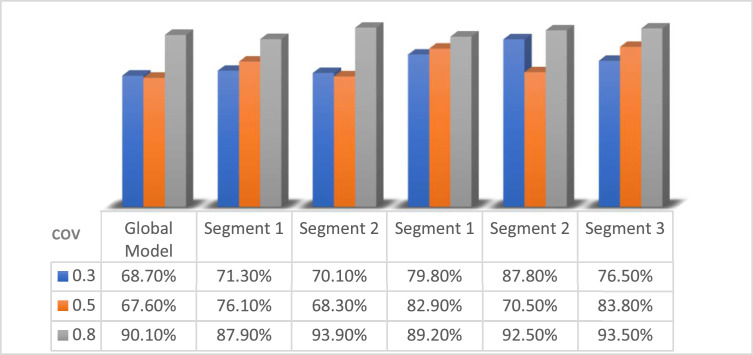
R^2^ values of the simulation study with *n* = 50.

**Fig. 5:**
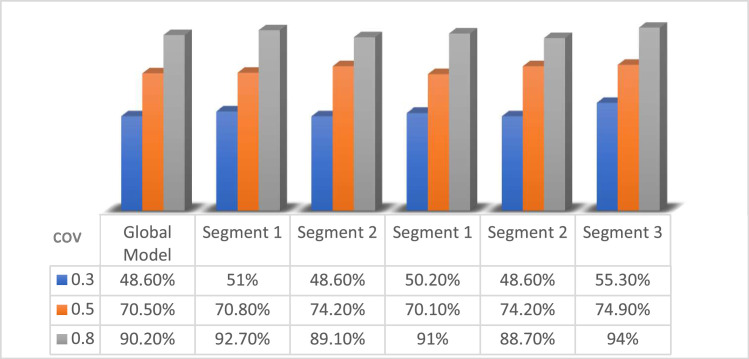
R^2^ values of the simulation study with *n*= 200.

**Fig. 6:**
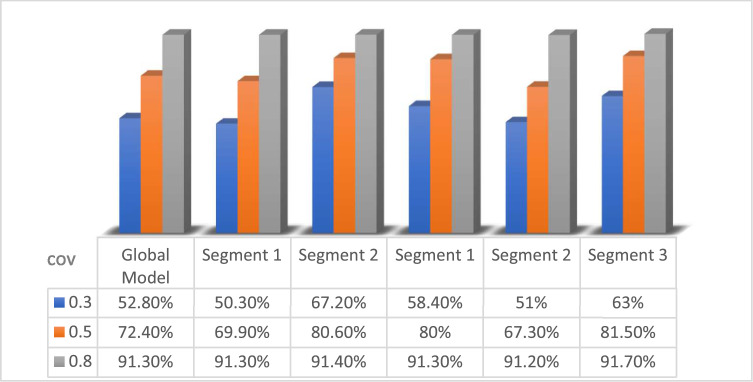
R^2^ values of the simulation study with *n* = 1000.

Model evaluation in the simulation study was carried out using the R^2^value of global model and the PLS SEM KKC local model. The following R^2^ values in the nine simulation study designs are presented in Table 8, Fig. 4, Fig. 5, Fig. 6:

**Table 8:** R^2^ Values of the simulation study with *n* = 50, *n* = 200, and *n* = 1000.

	Level covarian	Global Model	Simulation Study PLS SEM KKC				
			*k* = 2		*k* = 3		
			Segment 1 (S1K2)	Segment 2 (S2K2)	Segment 1 (S1K3)	Segment 2 (S2K3)	Segment 3 (S3K3)
*n* = 50	0.3	68.70 %	71.30 %	70.10 %	79.80 %	87.80 %	76.50 %
	0.5	67.60 %	76.10 %	68.30 %	82.90 %	70.50 %	83.80 %
	0.8	90.10 %	87.90 %	93.90 %	89.20 %	92.50 %	93.50 %

	Level covarian	Global Model	Simulation Study PLS SEM KKC				
			*k* = 2		*k* = 3		
			Segment 1 (S1K2)	Segment 2 (S2K2)	Segment 1 (S1K3)	Segment 2 (S2K3)	Segment 3 (S3K3)
*n* = 200	0.3	48.60 %	51 %	48.60 %	50.20 %	48.60 %	55.30 %
	0.5	70.50 %	70.80 %	74.20 %	70.10 %	74.20 %	74.90 %
	0.8	90.20 %	92.70 %	89.10 %	91 %	88.70 %	94 %

	Level covarian	Global Model	Simulation Study PLS SEM KKC				
			*k* = 2		*k* = 3		
			Segment 1 (S1K2)	Segment 2 (S2K2)	Segment 1 (S1K3)	Segment 2 (S2K3)	Segment 3 (S3K3)
*n* = 1000	0.3	52.80 %	50.30 %	67.20 %	58.40 %	51 %	63 %
	0.5	72.40 %	69.90 %	80.60 %	80 %	67.30 %	81.50 %
	0.8	91.30 %	91.30 %	91.40 %	91.30 %	91.20 %	91.70 %

Based on the simulation study results in Table 8 for small sample sizes (*n* = 50), it can be seen that the highest R^2^ values are consistently obtained at the covariance level of 0.8. R^2^ value in the global model for covariance 0.3 is 68.70 %, while for 0.5 it drops slightly to 67.60 %, and increases significantly to 90.10 % at covariance 0.8. Similarly, the SEM-PLS results of KKC with 2 clusters (S1K2 and S2K2) and 3 clusters (S1K3, S2K3, S3K3) generally show a sharp increase pattern as covariance increases. The simulation results on S1K2 show that the R^2^ value increases from 71.30 % at covariance level 0.3 to 87.90 % at covariance level 0.8. The simulation results at S2K2 show that the R^2^ value increases from 70.10 % at covariance level 0.3 to 93.90 % at covariance level 0.8. The simulation results at S1K3 show that the R^2^ value increases from 79.80 % at covariance level 0.3 to 89.20 % at covariance level 0.8. The simulation results at S2K3 show that the R^2^ value increases from 87.80 % at covariance level 0.3 to 92.50 % at covariance level 0.8. The simulation results at S3K3 show that the R^2^ value increases from 76.50 % at covariance level 0.3 to 93.50 % at covariance level 0.8. It shows that in small samples, the strength of the relationship between variables as indicated by the covariance value greatly affects the amount of variation that can be explained by the model. Therefore, for small sample sizes (*n* = 50), the higher the correlation between variables, the higher the accuracy of the model in explaining data variance.

Moreover, Table 8 shows the simulation study results for medium sample size (*n* = 200), there is a similar pattern with n = 50, but the variation between covariances is more stable and consistent. R^2^ value in the global model increased from 48.60 % at covariance level 0.3 to 70.50 % at covariance level 0.5 and significantly increased to 92.70 % at covariance level 0.8. The increase is also seen in the results of PLS SEM KKC with 2 clusters (S1K2 and S2K2) and 3 clusters (S1K3, S2K3, S3K3) generally showing a consistent pattern of increase as covariance increases. The simulation results on S1K2 show that R^2^ value increases from 51 % at covariance level 0.3 to 70.80 % at covariance level 0.5 and 92.70 % at covariance level 0.8. The simulation results on S2K2 showing that R^2^ value increases from 48.6 % at covariance level 0.3 to 74.20 % at covariance level 0.5 and 89.10 % at covariance level 0.8. The simulation results on S1K3 show that R^2^ value increases from 50.20 % at covariance level 0.3 to 70.10 % at covariance level 0.5 and 91 % at covariance level 0.8. The simulation results on S2K3 show that R^2^ value increases from 48.60 % at covariance level 0.3 to 74.20 % at covariance level 0.5 and 88.70 % at covariance level 0.8. The simulation results on S3K3 show that R^2^ value increases from 55.30 % at covariance level 0.3 to 74.90 % at covariance level 0.5 and 94 % at covariance level 0.8. With a medium sample size (*n* = 200), the variability of the data can be better captured by the model, so the effect of covariance is more measurable and the results become more stable. The medium sample sizes provide higher estimation stability than small sizes, but they still show significant covariance effects.

Additionally, based on the simulation study results in Table 8 for large sample sizes (*n* = 1000), R^2^ value still increases as the covariance increases, but the increase becomes more controllable and less volatile than for small samples. In the global model, R^2^ value increases from 52.80 % at covariance level 0.3 to 72.40 % at covariance level 0.5 and increases to 91.30 % at covariance level 0.8. The increase is also seen in the results of PLS SEM KKC with 2 clusters (S1K2 and S1K2) and 3 clusters (S1K3, S2K3, S3K3) generally showing a consistent pattern of increase as covariance increases. The simulation results on S1K2 show that R^2^ value increases from 50.30 % at covariance level 0.3 to 69.90 % at covariance level 0.5 and 91.30 % at covariance level 0.8. The simulation results on S2K2 show that R^2^ value increases from 67.20 % at covariance level 0.3 to 80.60 % at covariance level 0.5 and 91.40 % at covariance level 0.8. The simulation results on S1K3 show that R^2^ value increases from 58.40 % at covariance level 0.3 to 80 % at covariance level 0.5 and 91.30 % at covariance level 0.8. The simulation results on S2K3 show that R^2^ value increases from 51 % at covariance level 0.3 to 67.30 % at covariance level 0.5 and 91.20 % at covariance level 0.8. The simulation results on S3K3 show that R^2^ value increases from 63 % at covariance level 0.3 to 81.50 % at covariance level 0.5 and 91.70 % at covariance level 0.8. The simulation results with a large sample size (*n* = 1000) resulted in high stability to covariance variation and minimized the effect of small samples that could lead to overfitting or high variability. Thus, it can be stated that a large sample size can increase the reliability of modeling results and show the performance of the SEM PLS KKC model that is more consistent and robust to variations in correlation between variables.

## Limitations

The following are the limitations of this research:

1. The global PLS SEM model used only the Path weighting scheme. Alternative schemes like Centroid or Factorial were not explored and may yield different results.
2. Segmentation was limited to the RBF (Gaussian) kernel function. While effective for capturing non-linear patterns, other kernel types (e.g., polynomial, sigmoid) were not compared in this study.
3. The kernel parameter κ=0.5 based on σ=1. This fixed value is commonly used as a baseline and provides a moderate sensitivity to residual structure. However, it was not tuned for optimal performance, and future work should consider cross-validation or data-driven parameter selection.
4. The number of clusters was fixed at *k* = 2 and *k* = 3. Although this supports comparative analysis, adaptive cluster selection methods such as CH index may yield more optimal segmentation.
5. The PLS SEM KKC performed well for sample sizes up to *n* = 1000, its scalability may be limited for larger datasets. As the sample size increases, the residual structure may become more complex and harder to segment meaningfully. Future research could apply approximation techniques to improve scalability.

## Ethics statements

Data on students' behavioral intention to use e-learning is obtained based on primary data who are active users of e-learning at Universitas Muhammadiyah Sidoarjo, Indonesia.

## CRediT authorship contribution statement

**Cindy Cahyaning Astuti:** Conceptualization, Methodology, Data curation, Formal analysis, Software, Writing -original draft, Visualization. **B.W. Otok:** Conceptualization, Methodology, Writing - review & editing, Validation, Supervision. **S. Andari:** Methodology, Writing – review & editing, Validation, Supervision.

All authors finally revised the paper and approved for final submission.

## Related research article

None.

## For a published article

None.

## Supplementary material *and/or* additional information [OPTIONAL]

None.

## Declaration of competing interest

The authors declare that they have no known competing financial interests or personal relationships that could have appeared to influence the work reported in this paper.

## Figures and Tables

**Table A1:** Path coefficients global PLS SEM and PLS SEM KKC.

Simulation Design	Variable	Global PLS SEM				PLS SEM KKC (*k* = 2)			PLS SEM KKC (*k* = 3)		
				Segment 1			Segment 2	Segment 1		Segment 2	Segment 3
Simulation 1	PE		0.288		0.343		0.275	0.274		0.342	0.227
	EE	0.254		0.199			0.357	0.138		0.406	0.267
	SI	0.294		0.242			0.288	0.590		0.312	-0.014
	FC	0.245		0.274			0.230	0.149		0.147	0.565
Simulation 2	PE	0.217		0.303			0.151	0.209		0.218	0.222
	EE	0.116		0.078			0.118	-0.041		0.141	0.167
	SI	0.249		0.293			0.221	0.247		0.262	0.284
	FC	0.289		0.231			0.357	0.467		0.223	0.267
Simulation 3	PE	0.268		0.257			0.279	0.303		0.253	0.298
	EE	0.250		0.261			0.240	0.245		0.252	0.294
	SI	0.179		0.179			0.213	0.191		0.168	0.205
	FC	0.201		0.184			0.267	0.206		0.212	0.179
Simulation 4	PE	0.371		0.582			0.343	0.429		0.149	0.295
	EE	0.229		0.238			0.411	0.651		0.633	-0.360
	SI	0.229		0.145			-0.156	-0.139		0.058	1.052
	FC	0.072		-0.043			0.389	0.029		0.043	-0.168
Simulation 5	PE	0.277		0.218			0.329	0.381		0.227	0.336
	EE	0.245		0.285			0.224	0.124		0.294	0.311
	SI	0.112		0.151			0.075	0.124		0.042	0.059
	FC	0.307		0.289			0.330	0.291		0.395	0.264
Simulation 6	PE	0.193		0.178			0.236	0.227		0.168	0.237
	EE	0.310		0.332			0.245	0.263		0.320	0.341
	SI	0.179		0.178			0.183	0.188		0.178	0.175
	FC	0.271		0.248			0.339	0.313		0.258	0.255
Simulation 7	PE	0.399		0.521			0.274	-0.014		0.360	0.777
	EE	0.118		0.156			0.015	0.236		-0.007	-0.006
	SI	0.250		0.152			0.448	0.232		0.403	0.123
	FC	0.212		0.133			0.263	0.522		0.231	0.089
Simulation 8	PE	0.280		0.305			0.252	0.440		0.161	0.297
	EE	0.329		0.335			0.328	0.212		0.404	0.352
	SI	0.129		0.205			0.087	0.169		-0.016	0.254
	FC	0.244		0.149			0.311	0.174		0.414	0.101
Simulation 9	PE	0.288		0.292			0.281	0.293		0.291	0.276
	EE	0.279		0.263			0.298	0.259		0.274	0.295
	SI	0.148		0.149			0.149	0.119		0.166	0.163
	FC	0.272		0.283			0.259	0.316		0.255	0.254

**Table A2:** Signifikansi global PLS SEM and PLS SEM KKC on simulation study.

Simulation Design	Variable		Global PLS SEM	PLS SEM KKC (*k* = 2)			PLS SEM KKC (*k* = 3)		
				Segment 1	Segment 2	Segment 1		Segment 2	Segment 3
Simulation 1	PE	0.018*		0.058	0.171	0.313		0.181	0.332
	EE	0.004*		0.150	0.049*	0.565		0.044*	0.181
	SI	0.001*		0.099	0.077	0.003*		0.140	0.937
	FC	0.013*		0.028*	0.224	0.522		0.543	0.005*
Simulation 2	PE	0.001*		0.000*	0.089	0.082		0.023	0.020*
	EE	0.154		0.446	0.210	0.745		0.230	0.114
	SI	0.000*		0.000*	0.063	0.057		0.015	0.007*
	FC	0.000*		0.012*	0.000*	0.000*		0.043	0.024*
Simulation 3	PE	0.000*		0.000*	0.000*	0.000*		0.000*	0.000*
	EE	0.000*		0.000*	0.000*	0.000*		0.000*	0.000*
	SI	0.000*		0.000*	0.000*	0.000*		0.000*	0.000*
	FC	0.000*		0.000*	0.000*	0.000*		0.000*	0.004*
Simulation 4	PE	0.000*		0.002*	0.103	0.228		0.624	0.284
	EE	0.066		0.207	0.126	0.045*		0.252	0.294
	SI	0.212		0.557	0.626	0.641		0.914	0.028
	FC	0.570		0.836	0.059	0.924		0.912	0.567
Simulation 5	PE	0.000*		0.016	0.000*	0.004		0.021	0.000
	EE	0.000*		0.001*	0.002	0.283		0.001	0.006
	SI	0.077		0.117	0.324	0.308		0.701	0.574
	FC	0.000*		0.002*	0.000*	0.010		0.000	0.005
Simulation 6	PE	0.000*		0.000*	0.000*	0.000*		0.000*	0.000*
	EE	0.000*		0.000*	0.000*	0.000*		0.000*	0.000*
	SI	0.000*		0.000*	0.000*	0.000*		0.000*	0.000*
	FC	0.000*		0.000*	0.000*	0.000*		0.000*	0.000*
Simulation 7	PE		0.012	0.059	0.292	0.981		0.316	0.046
	EE		0.319	0.429	0.941	0.474		0.978	0.986
	SI		0.083	0.444	0.036	0.537		0.328	0.589
	FC		0.029	0.499	0.104	0.124		0.307	0.757
Simulation 8	PE		0.000*	0.000*	0.000*	0.007*		0.089	0.000*
	EE		0.000*	0.000*	0.001*	0.136		0.001*	0.000*
	SI		0.018*	0.001*	0.237	0.071		0.867	0.000*
	FC		0.000*	0.077	0.000*	0.078		0.001*	0.215*
Simulation 9	PE		0.000*	0.000*	0.000*	0.000*		0.000*	0.000*
	EE		0.000*	0.000*	0.000*	0.000*		0.000*	0.000*
	SI		0.000*	0.000*	0.000*	0.009*		0.000*	0.000*
	FC		0.000*	0.000*	0.000*	0.000*		0.000*	0.000*

Significant at α = 5 %

## Data Availability

Data will be made available on request.
